# Abstracts

**DOI:** 10.1155/S146392468200039X

**Published:** 1982


### Abstracts

### Page 161

### Bulk pharmaceutical research data management

H. B. Woodruff *et al.*

Modern analytical instrumentation can generate huge amounts of data in a short period of time. Usually the data are hand-transcribed onto an analytical form and filed away, often making sample tracking a very tedious and time-consuming operation. Computerizing the storage and retrieval of analytical results can eliminate some of these problems; however, it is crucial that the software employed is both friendly and tolerant of user errors. A package of computer programs which places top priority on human engineering and the human interface has been developed and implemented in the Analytical Research Department of Merck, Sharp & Dohme Research Laboratories. These programs have proven to constitute a convenient data management package that has been accepted well by laboratory personnel.

### Approche à l'informatisation de la gestion des données en chimie analytique

H. B. Woodruff *et al.*

Les instruments d'analyses modernes peuvent générer d'énormes volumes de données dans une courte période de temps. Habituellement, les données sont transcrites manuellement sur un compte rendu analytique, puis elles sont rangées, ceci créant pour la recherche ultérieure d'un échantillon, une opération difficile et longue. L'informatisation de l'archivage et de la recherche des résultats analytiques peut éliminer quelques-uns de ces problèmes; cependant, il est important que le logiciel employé soit à la fois bienveillant et tolérant, vis-à-vis des erreurs des utilisateurs. Un ensemble de programmes pour ordinateur qui a pour premier objectif de faciliter cette interface homme-machine a été développé et installé dans les laboratoires de l'Analytical Research Department of Merck, Sharp & Dohme Research Laboratories. Ces programmes ont montré qu'ils constituaient un ensemble valable pour la gestion des données et qu'ils étaient bien acceptés par le personnel de laboratoire.

### Ein Versuch zur computerisierten Datenverarbeitung im analytischen Labor

H. B. Woodruff *et al.*

Moderne analytische Instrumentierung kann innerhalb kurzer Zeit riesige Berge von Daten generieren. Normalerweise werden Daten von Hand auf ein analytisches Formular übertragen und dann abgelegt. Das Wiederauffinden von Daten wird dadurch häufig mühsam und zeitraubend. Ein computerisiertes Ablegen und Hervorholen analytischer Resultate kann einige dieser Probleme beseitigen. Es ist jedoch entscheidend, dass die verwendete Software benützerfreundlich ist und Fehler des Benützers toleriert. Im Analytical Research Department von Merck, Sharp & Dohme Research Laboratories wurde ein Paket von Computer-Programmen entwickelt und in Betrieb genommen, welches erste Priorität auf die menschlichen Aspekte und das Zusammenspiel mit dem Benützer legt. Diese Programme stellten sich als ein bequemes Paket für die Verarbeitung von Daten heraus, das vom Laborpersonal gut akzeptiert wurde.

---

### Page 165

### Desk-top computer in the clinical laboratory linked to automatic multichannel biochemistry analysers

L. Funding and Y. Bergqvist

This paper describes an interactive desk-top microcomputer system connected on line to SMA II (Technicon) and G-300 (Greiner) analysers. The system contains various program modules, written in BASIC, to generate quality-control statistics, administrative statistics and retrieval of patients' results. Self-adhesive labels are used by the system to tabulate patients' results. The system is designed to be used by staff who are not familiar with computers.

### Mini-ordinateur dans le laboratoire clinique en combinaison avec analysateur multi-canal biochimique

L. Funding and Y. Bergqvist

Ce travail décrit un système interactif avec mini-ordinateur connecté à un analysateur SMA II (Technicon) et G-300 (Greiner). Le système contient de différents programmes écrits en BASIC qui effectuent la statistique du contrôle de qualité, l'administration ainsi que la recherche des résultats concernants les différents malades. Pour les tableaux des résultats des malades des étiquettes autocollantes sont utilisées. Le système est conçu de façon qu'il peut être utilisé, sans problèmes, d'un personnel sans expérience avec ordinateurs.

### Tischrechner im klinischen Laboratorium im Verband mit biochemischen Vielkanal-Analysatoren

L. Funding and Y. Bergqvist

Die Arbeit beschreibt ein interaktives Tischrechnersystem mit angeschlossenen SMA II (Technicon) und G-300 (Greiner) Analysatoren. Das System enthält verschiedene, in BASIC geschriebene Programm-Module, die die Statistik der Qualitätskontrolle und der Administration sowie das Heraussuchen von Resultaten zu den Patienten durchführen. Zur Tabellierung der Patienten-Resultate werden Selbstklebeetiketten verwendet. Das System ist so aufgebaut, dass es mühelos von Personal benützt werden kann, das keine Erfahrung im Umgang mit einem Computer hat.

---

### Page 169

### Sample and data random management in a medical laboratory equipped with automatic analysers

G. Barbaresi *et al.*

The authors describe a local information system which is based on a minicomputer on-line with automatic analysers. Positive identification of samples is performed by means of an automatic optical reader. Information exchange between the clinical laboratory and the hospital data-processing centre (DPC) is permitted by data transfer on floppy disc. The system allows 'personalized' clinical reports to be printed out in the laboratory using patient census data obtained from the DPC by means of a floppy disc. On-line acquisition of analytical results from automatic analysers (an SMAC for plasma samples and a CLINILAB for urinalysis) are linked with their respective requests and patient data. The authors have achieved random management of samples arriving in their laboratory and automatic instrument analytical output. The system is shown to be cheap, versatile and easy to operate.

### Traitement de données et d'échantillons dans un laboratoire d'analyses médicales équipé d'analyseurs automatiques

G. Barbaresi *et al.*

Les auteurs décrivent un système informatique décentralisé basé sur un minicomputer branché en 'on-line' avec des analyseurs automatiques. L'identification automatique des échantillons est obtenue aux moyens d'un lecteur optique. Les échanges d'informations entre le laboratoire et l'ordinateur central de l'hopital sont réalisés à l'aide de transferts de floppy disc. Le système permet la sortie dans le laboratoire de compte rendus 'personnalisés', en utilisant les données d'identification du patient obtenues de l'ordinateur central à l'aide de transferts de floppy disc. Les résultats analytiques obtenus 'on-line' des différents analyseurs automatiques (un SMAC pour les échantillons de sang et un CLINILAB pour les prélèvements d'urine) sont associés aux demandes et identification du patient. Les auteurs ont ainsi mis en place un traitement d'organisation des échantillons arrivant dans le laboratoire et des résultats provenant des analyseurs automatiques. Le système ainsi décrit semble être bon marché, flexible et facile à utiliser.

### Organisation und Verwaltung von Proben und Daten in einem mit automatischen Analysatoren ausgerüsteten medizinischen Labor
G. Barbaresi *et al.*

Die Autoren beschreiben ein lokal aufgebautes Informations system, das auf einem Minicomputer mit on-line angeschlossenen automatischen Analysatoren beruht. Positive Probenidetifikation wird mit Hilfe eines automatischen Lesers durchgeführt. Datenaustausch zwischen dem klinischen Labor und dem Spital-Datenverarbeitungszentrum geschieht mit Hilfe des Transfers von Daten auf Floppy Disk. Das System ermöglicht das Ausdrucken 'persönlicher' klinischer Berichte im Laboratorium unter Benützung der Personalien, die vom Datenverarbeitungszentrum auf einer Floppy Disk geliefert werden. Die on-line Erfassung analytischer Resultate von den automatischen Analysatoren (ein SMAC für die Plasma-Proben und ein CLINILAB für die Harnstoffanalyse) wird mit den Patientendaten verbunden und an die jeweilige Anfragestelle zurückgeführt. Die Autoren erreichten damit eine Verwaltung der Proben in ihrem Laboratorium in beliebiger Reihenfolge mit gleichzeitig automatischer Erfassung der Daten der analytischen Instrumente. Es wird gezeigt, dass das System billig, flexibel und leicht zu benützen ist.

---

### Page 174

### Desk-top computer as interface and terminal in the clinical laboratory
J. I. Dydula *et al.*

The clinical laboratory utilizes many different analytical principles and equipment of different degrees of mechanization. Consequently, the analysers are equipped with data-output devices of many configurations that seldom fit into the input devices of a given large computer. A desk-top computer (DTC) provides a flexible interface, permitting two-way communication between analyser, technician, and central computer. The advantages of the DTC are capture and transfer of different types of data, introduction of different kinds of specimen identification, correction of errors in specimen identification and results, quality control at the work station, data storage during central computer break-down, and a possibility of software exchanges by the user. A set of useful requirements for a DTC is presented, comprising input/output facilities, hardware features, memory, programming language, program library, instructions, source and support, availability, and uniformity. Practical examples of implementation are given, using an APPLE II as an interface.

### Un minicomputer comme interface et station d'entrée et de sortie dans le laboratoire clinique
J. I. Dydula *et al.*

Le laboratoire clinique utilise de différentes méthodes analytiques et des instruments de degrés de mécanisation différents. Les analysateurs ont des circuits de sortie de données des plus diverses configurations qui, le plus souvent, ne sont pas compatibles avec les circuits d'entrée des grandes calculatrices utilisées. Un petit ordinateur représente un interface flexible qui permet la communication dans les deux senses entre l'analysateur, l'utilisateur et la calculatrice centrale. Les avantages de la calculatrice sont la réception et le transfert de diverses sortes de données, la réception de diverses sortes d'identifications d'échantillons, la correction d'erreurs dans l'identification d'échantillons et dans les résultats, le contrôle de qualité au lieu de travail, la mémorisation de données pendant une panne de la calculatrice centrale et la possibilité de modifier le software par l'utilisateur. Une liste des éxigences utiles posée à une calculatrice est présentée. Elle comprend les possibilités d'entrée et de sortie, les propriétés du hardware, les mémoires, la langue de programmation, la bibliothèque de programme, les instructions, le fournisseur, le service après-vente, les possibilités d'achat et l'uniformité. Des exemples pratiques sont présentés qui utilisent le APPLE II comme interface.

### Tischrechner als Interface und Terminal im klinischen Laboratorium
J. I. Dydula *et al.*

Das klinische Labor verwendet viele verschiedene analytische Methoden und Geräte mit verschiedenem Mechanisie-Rungsgrad. Entsprechend sind die Analysatoren mit Datenausgangsschaltungen verschiedenster Konfigurationen versehen, die selten auf die Eingangsschaltung eines vorhandenen Grossrechners passen. Ein Tischrechner stellt ein flexibles Interface dar, das Zweiwegkommunikationen zwischen Analysator, Benützer und Zentralrechner ermöglicht. Die Vorteile der Tischrechner sind Erfassung und Transfer verschiedener Arten von Daten, Uebernahme verschiedener Arten von Probenidentifikation, Korrektur von Fehlern in der Probenidentifikation und in den Resultaten, Qualitätskontrolle am Arbeitsplatz, Datenspeicherung während eines Ausfalls des Zentralrechners und Möglichkeit der Aenderung der Software durch den Benützer. Eine Liste mit nützlichen Anforderungen an den Tischrechner wird vorgestellt, die Ein-/Ausgangsmöglichkeiten, Hardware-Eigenschaften, Speicher, Programmiersprache, Programmbibliothek, Instruktionen, Lieferant und Unterstützung, Erhältlichkeit und Einheitlichkeit umfasst. Praktische Beispiele werden aufgeführt, die einen APPLE II als Interface benützen.

---

### Page 176

### Titration of spoilt beer samples by flow-injection analysis
J. G. Williams *et al.*

Beers that are spoilt during manufacture, or in the course of distribution to the public, are the subject of a repayment of alcohol excise duty. The spoilt samples are returned from the distribution network to the brewery of origin as unfit for consumption. The Laboratory of the Government Chemist measures the extent of microbiological oxidation of the alcohol in the beer to acetic acid. A flow-injection system has been designed for automating the colorimetric titration of the acetic acid with sodium hydroxide solution. A circuit is described for initiating and terminating monitoring by a PET microcomputer of the real-time separation of the inflection points of the titration curves. The circuit employs both the first and second derivatives of the change of transmittance with time to control the output of an AND gate. A program written in BASIC is given for utilizing the output from the AND gate and producing a simultaneous linear regression analysis of the results. The effects of sample viscosity and colour on colorimetric flow-injection titrations are also discussed.

### Titrage d'échantillons de bière gâtée par l'analyse par injection continue

J. G. Williams *et al.*

La bière qui se gâte lors de la production ou au cours de la distribution, donne droite à un remboursement des impôts sur l'alcool. Les échantillons gâtés sont renvoyés à la brasserie avec la remarque 'imbuvable'. Le 'Laboratory of the Government Chemist' mesure l'oxydation microbiologique de l'alcool dans la bière en acide acétique. Un système à injection continue (Flow Injection Analysis) a été installé pour permettre le titrage colorimétrique de l'acide acétique avec la soude de façon automatique. Un circuit est décrit qui permet le contrôle de la séparation des points d'inflection des courbes de titrage avec un PET micro-computer. Le circuit utilise la première et la deuxième dérivée de la transmission par rapport au temps pour contrôler la sortie d'une porte AND. Un programme écrit en BASIC utilise la sortie de la porte AND et produit une analyse de régression linéaire simultanée des résultats. L'influence de la viscosité de l'échantillon et de sa couleur sur le titrage colorimétrique par injection est également discutée.

### Titration von Proben verdorbenen Biers mit der kontinuierlichen Injektions-Analyse

J. G. Williams *et al.*

Bier, das während der Herstellung oder im Laufe der Verteilung an die Oeffentlichkeit verdirbt, ist Gegenstand einer Rückvergütung der Alkoholsteuer. Die verdorbenen Proben werden aus dem Verteilernetz an die Hersteller-Brauerei mit der Bemerkung 'ungeniessbar' retourniert. Das Laboratory of the Government Chemist misst das Ausmass der mikrobiologischen Oxidation des Alkohols in Bier zu Essigsäure. Ein kontinuierliches Injektionssystem (Flow Injection Analysis) wurde so aufgebaut, dass die kolorimetrische Titration der Essigsäure mit Natronlauge automatisch durchgeführt werden kann. Eine Schaltung ist beschrieben, die die Ueberwachung der Trennung der Wendepunkte der Titrationskurven mit einem PET Mikrocomputer injiziert und terminiert. Die Schaltung verwendet die erste und die zweite Ableitung der Transmission in Bezug auf die Zeit, um den Ausgang eines AND-Tors zu steuern. Ein in BASIC geschriebenes Programm benützt den Ausgang des AND-Tors und produziert eine simultane lineare Regressionsanalyse der Resultate. Der Einfluss der Probenviskosität und Farbe auf die kolorimetrische Injektionstitration ist ebenfalls diskutiert.

---

### Page 183

### The measurement of erythrocyte transketolase activity on a discrete analyser

C. R. Milner *et al.*

A manual kinetic method for erythrocyte transketolase (TK) was modified for automation on a discrete analyser (Gilford System 5), thus making it a simple, economical and convenient method.

The TK and thiamine-activated TK activities were measured by both methods on 51 specimens. For the manual and automated methods, the TK activities had a mean of 0·81 and 0·86 U/g Hb (S.D.s were 0·25 and 0·26) respectively, while for the activated-TK activities the mean values were 0·94 and 0·98 U/g Hb (S.D.s were 0·25 and 0·27) respectively. The two methods gave a correlation coefficient of 0·87 for the TK activity and 0·89 for the activated-TK activity. The within-day precision was good, with a C.V. of 7·4% at low activity and a C.V. of 5·0% at high activity.

The reference range for our automated TK method was 0·6 to 1·3 U/g Hb and the thiamine pyrophosphate (TPP) effect ranged from 0–25%.

### Mesure de l'activité transcétolasique érythrocytaire à l'aide d'un analyseur discret

C. R. Milner *et al.*

Une méthode manuelle cinétique pour le dosage de la transcétolase érythrocytaire a été modifiée pour être automatisé sur un analyseur discret (Gilford System 5), le système analytique ainsi défini est simple et économique.

Les activités de transcétolase (TK) et de transcétolase activée à la thiamine sont mesurées par deux méthodes sur 51 spécimens. Pour les méthodes manuelles et automatiques, les activités TK ont une moyenne de 0,81 et 0,86 U/g Hb (S.D. à 0,25 et 0,26) respectivement, alors que les activités de TK activée ont des valeurs moyennes de 0,94 et 0,98 U/g Hb (S.D. 0,25 et 0,27) respectivement. Les deux méthodes donnent des coefficients de corrélation de 0,87 pour l'activité TK et de 0,89 pour l'activité TK activée. La répétabilité est bonne avec un coefficient de corrélation à 7,4% pour des activités basses et à 5% pour des activités élevées.

L'intervalle de référence pour notre méthode automatisée de TK est de 0,6 à 1,3 U/g Hb et l'activité par la thiamine pyrophosphate (TPP) augmente les valeurs de l'intervalle de référence jusqu'à 25%.

### Die Bestimmung von Erythrozyten-Transketolasenaktivität mit einem diskreten Analysator

C. R. Milner *et al.*

Eine manuelle kinetische Methode für Erythrozyten-Transketolase (TK) wurde für Automation auf einem diskret arbeitenden Analysator (Gilford System 5) modifiziert. Damit entstand daraus eine einfache, ökonomische und bequeme Methode.

TK und Thiamin aktivierte TK-Aktivitäten wurden mit beiden Methoden an 51 Proben bestimmt. Für die manuelle und automatische Methode betrugen die Mittelwerte der TK-Aktivitäten 0,81 bzw. 0,86 U/g (Standardabweichung 0,25 bzw. 0,26), während für die aktivierten TK-Aktivitäten die Mittelwerte 0,94 bzw. 0,98 U/g Hb (Standardabweichung 0,25 bzw. 0,27) betrugen. Die beiden Methoden ergaben einen Korrelationskoeffizienten von 0,87 für die TK-Aktivität und 0,89 für die aktivierte TK-Aktivität. Die Präzision innerhalb eines Tages war gut, mit einem C.V. von 7,4% bei niedriger Aktivität und einem C.V. von 5,0% bei hoher Aktivität.

Der Bezugsbereich für unsere automatisierte TK-Methode betrug 0,6–1,3 U/g Hb und die Thiaminpyrophosphat-Wirkung überstrich den Bereich von 0–25%.

### Page 186

### An improved flow-through phototransducer

T. J. Sly *et al.*

An improved flow-through photometric detector has been developed, based on the design of Betteridge *et al.* The new unit is easier to construct and is more reliable and robust than the original design, whilst offering superior resolution. A revised flowcell has also been developed, with an effective volume of ca. 0·8 $\mu$l and a path length of 1 mm. Output from the unit is in the form of an analogue voltage which is linear with absorbance.

### Un capteur optique perfectionné pour la mesure de flux

T. J. Sly *et al.*

Un capteur optique perfectionné pour la mesure de flux a été développé suivant le prototype de Betteridge et collaborateurs. La nouvelle unité est plus simple à construire, plus sûre et plus robuste que le prototype original, tout en offrant une résolution supérieure. Une cuve à circulation a aussi été développée, avec un volume efficace de 0,8 $\mu$l et un trajet optique de 1 mm. Le signal sortant de cette unité est analogique et linéaire avec l'absorbance.

### Verbesserte Durchflusszelle für photometrische Detektion

T. J. Sly *et al.*

Eine verbesserte Durchflusszelle für photometrische Detektion wird vorgestellt, die von der Konstruktion von Betteridge *et al.* ausgeht. Die neue Einheit ist viel einfacher zu bauen und ist bei überlegener Auflösung zudem zuverlässiger und robuster als die ursprüngliche Konstruktion. Ferner wurde eine Durchflusszelle auf ein effektives Volumen von ca. 0·8 $\mu$l und eine Weglänge von 1 mm modifiziert. Das Ausgangssignal der Einheit ist eine Analogspannung, die linear zur Absorption ist.

### Notes for Authors

*Journal of Automatic Chemistry* covers all aspects of automation and mechanization in analytical, clinical and industrial environments. The *Journal* publishes original research papers; short communications on innovations, techniques and instrumentation, or current research in progress; reports on recent commercial developments; and meeting reports, book reviews and information on forthcoming events. All research papers are refereed.

### Manuscripts

Two copies of articles should be submitted to the Editor. All articles should be typed in double spacing with ample margins, on one side of the paper only. The following items should be sent: (1) a title-page including a brief and informative title, avoiding the word 'new' and its synonyms; a full list of authors with their affiliations and full addresses; (2) an abstract of about 250 words—this should succinctly describe the scope of the contribution and highlight significant findings or innovations; it should be written in a style which can easily be translated into French and German; (3) the main text with sections and subsections numbered; (4) appendices (if any); (5) references; (6) tables, each table on a separate sheet and accompanied by a caption; (7) illustrations (diagrams, drawings and photographs) numbered in a single sequence from 1 upwards and with the author's name on the back of every illustration; captions to illustrations should be typed on a separate sheet.

### References

References should be indicated in the text by numbers following the author's name, i.e. Skeggs [6]. In the reference section they should be arranged thus.

to a journal

Manka, D. P., *Journal of Automatic Chemistry*, **3** (1981), 119.

to a book

Malmstadt, H. V., in *Topics in Automatic Chemistry*, Ed. Stockwell, P. B. and Foreman, J. K. (Horwood, Chichester, 1978), p. 68.

### Illustrations

Line diagrams are preferred to photographs. Original copies of diagrams and drawings should be supplied and should be drawn to be suitable for reduction to the page or column width of the *Journal*, i.e. 85 mm or 179 mm, with special attention to lettering size. Photographs may be sent as glossy prints or as negatives.

### Proofs and offprints

The principal or corresponding author will be sent galley proofs for checking and will receive 50 offprints free of charge. Additional offprints may be ordered on a form which accompanies the proofs.

*Manuscripts should be sent to the Editor: Dr Peter B. Stockwell, Plasma-Therm Ltd, Unit 3, 2/3 Kangley Bridge Road, Lower Sydenham, London SE26 5AR.*

